# Association of plasma somatostatin with disease severity and progression in patients with autosomal dominant polycystic kidney disease

**DOI:** 10.1186/s12882-018-1176-y

**Published:** 2018-12-19

**Authors:** A. Lianne Messchendorp, Edwin M. Spithoven, Niek F. Casteleijn, Wendy A. Dam, Jacob van den Born, Wouter F. Tonnis, Carlo A. J. M. Gaillard, Esther Meijer, on behalf of the DIPAK Consortium, Joost Drenth, Johan W. de Fijter, Ron T. Gansevoort, Dorien J. M. Peters, Jack F. M. Wetzels, Robert Zietse

**Affiliations:** 1 Department of Nephrology, University Medical Center Groningen, University of Groningen, Groningen, The Netherlands; 2 Department of Urology, University Medical Center Groningen, University of Groningen, Groningen, The Netherlands; 3 Department of Pharmaceutical Technology and Biopharmacy, University of Groningen, Groningen, The Netherlands; 4 Division of Internal Medicine and Dermatology, University Medical Center Utrecht, University of Utrecht, Utrecht, The Netherlands

**Keywords:** ADPKD, Somatostatin, Biomarker, cAMP, Disease progression

## Abstract

**Background:**

Somatostatin (SST) inhibits intracellular cyclic adenosine monophosphate (cAMP) production and thus may modify cyst formation in autosomal dominant polycystic kidney disease (ADPKD). We investigated whether endogenous plasma SST concentration is associated with disease severity and progression in patients with ADPKD, and whether plasma SST concentrations change during treatment with a vasopressin V2 receptor antagonist or SST analogue.

**Methods:**

In this observational study, fasting concentrations of SST were measured in 127 ADPKD patients (diagnosed upon the revised Ravine criteria) by ELISA. cAMP was measured in 24 h urine by Radio Immuno Assay. Kidney function was measured (mGFR) as ^125^I-iothalamate clearance, and total kidney volume was measured by MRI volumetry and adjusted for height (htTKV). Disease progression was expressed as annual change in mGFR and htTKV. Additionally, baseline versus follow-up SST concentrations were compared in ADPKD patients during vasopressin V2 receptor antagonist (tolvaptan) (*n* = 27) or SST analogue (lanreotide) treatment (*n* = 25).

**Results:**

In 127 ADPKD patients, 41 ± 11 years, 44% female, eGFR 73 ± 32 ml/min/1.73m^2^, mGFR 75 ± 32 ml/min/1.73m^2^ and htTKV 826 (521–1297) ml/m, SST concentration was 48.5 (34.3–77.8) pg/ml. At baseline, SST was associated with urinary cAMP, mGFR and htTKV (*p* = 0.02, *p* = 0.004 and *p* = 0.02, respectively), but these associations lost significance after adjustment for age and sex or protein intake (*p* = 0.09, *p* = 0.06 and *p* = 0.15 respectively). Baseline SST was not associated with annual change in mGFR, or htTKV during follow-up (st. β = − 0.02, *p* = 0.87 and st. β = − 0.07, *p* = 0.54 respectively). During treatment with tolvaptan SST levels remained stable 38.2 (23.8–70.7) pg/mL vs. 39.8 (31.2–58.5) pg/mL, *p* = 0.85), whereas SST levels decreased significantly during treatment with lanreotide (42.5 (33.2–55.0) pg/ml vs. 29.3 (24.8–37.6), *p* = 0.008).

**Conclusions:**

Fasting plasma SST concentration is not associated with disease severity or progression in patients with ADPKD. Treatment with lanreotide caused a decrease in SST concentration. These data suggest that plasma SST cannot be used as a biomarker to assess prognosis in ADPKD, but leave the possibility open that change in SST concentration during lanreotide treatment may reflect therapy efficacy.

**Electronic supplementary material:**

The online version of this article (10.1186/s12882-018-1176-y) contains supplementary material, which is available to authorized users.

## Background

Autosomal dominant polycystic kidney disease (ADPKD) is characterized by progressive cyst formation in both kidneys, leading to kidney enlargement and loss of renal function [1]. On a cellular level the disease is characterized by dysregulated calcium influx in tubular epithelial cells and increased intracellular levels of cyclic adenosine monophosfate (cAMP). In turn, cAMP stimulates cellular proliferation and dedifferentiation, and causes fluid transport. These processes lead to dilation of tubules and to cyst formation [2].

Somatostatin (SST) is a naturally occurring hormone secreted mainly by cells of the nervous system, gastrointestinal tract and pancreatic islets. SST has an inhibitory effect on the release of growth hormone, pancreatic enzymes, and gastrointestinal peptides. In the kidney SST has multiple effects including inhibition of cAMP production [3] and inhibition of proliferation of renal cells [4–6]. It is therefore hypothesized that SST may have favorable effects in ADPKD. Indeed, randomized clinical trials suggest that systemic administration of SST analogues may be beneficial in slowing disease progression in ADPKD [7–9].

Given these findings, we hypothesized that endogenous, systemic SST levels are involved in the pathophysiologic cascade of ADPKD and therefore is associated with urinary cAMP, disease severity and progression. Furthermore, we hypothesized that administration of SST analogues may down-regulate systemic SST, and that the degree of systemic SST down-regulation may reflect therapy efficacy. Furthermore, we hypothesized that SST levels may change during treatment with a vasopressin V2 receptor antagonist since this treatment effectively inhibits cyst formation by suppressing cAMP levels [10, 11]. We therefore investigated in subjects with ADPKD whether fasting plasma concentration of endogenous SST is associated with 1) urinary cAMP excretion 2) disease severity and 3) disease progression, and 4) whether plasma SST concentration changes on treatment with the vasopressin V2 receptor antagonist tolvaptan or the SST analogue lanreotide.

## Material and methods

### Setting and subjects

For the first part of this study, all consecutive patients with ADPKD visiting our out-patient clinic from January 2007 until September 2012 were asked to participate. The diagnosis of APDKD was made based upon the revised Ravine criteria [12]. Subjects were considered ineligible in case they received renal replacement therapy, had undergone renal surgery, were unable to undergo MR imaging, had other medical conditions, systemic diseases or treatments potentially affecting renal function (such as pregnancy, lactation, diabetes mellitus, chronic use of NSAIDs).

For the second part of this study, plasma SST was measured in 2 additional groups of ADPKD patients, who participated in a clinical trial investigating a vasopressin receptor antagonist (*n* = 27) [13] and who participated in a clinical trial using a somatostatin analogue (*n* = 25) [14].

This study was performed in adherence to the declaration of Helsinki and all participants gave written informed consent allowing to use data for additional analysis. The institutional review board of the University Medical Center Groningen deemed this study exempt of assessment because of its post-hoc exploratory nature.

### Data collection and measurements

For the first part of our study, all ADPKD patients collected a 24 h urine 1 day prior to the baseline visit. At baseline blood pressure was assessed at rest in a supine position with a semi-automatic, non-invasive sphygmomanometer (Dinamap) for 15 min and height and weight were measured for the calculation of BMI and BSA (according to the Dubois formula) [15]. Next, fasting blood samples were drawn for the measurement of SST and creatinine and for *PKD* mutation analyses. For the measurement of GFR (mGFR), patients underwent a renal function measurement by the constant infusion method with ^125^I-Iothalamate at baseline and follow-up. MRI imaging was performed, both at baseline and follow-up for the measurement of TKV, using a standardized abdominal MRI imaging protocol with a 1.5-Tesla MR scanner (Magneto Avanto, Siemens, Erlangen, Germany) and with a 3.0 Tesla MR scanner (and Intera, Philips, Eindhoven, the Netherlands). We observed no differences in quality, i.e. suitability for TKV measurement, of images between both scanners in a previous study. We also observed no differences in TKV measured on T1 versus T2 weighted images between both scanners [16]. Therefore TKV measured on images of both MR scanners is comparable. Of note, a subset of patients (*n* = 26) underwent an extra blood draw during a follow-up of 6 weeks for the measurement of SST to study the stability of plasma SST concentration over time.

We additionally measured SST in patients who participated in two clinical studies. Protocols of these studies are described elsewhere [13, 14]. In short, patients were invited for a baseline visit receiving standard care. Next, patients were invited for a follow-up visit after 3 weeks to 3 months receiving either tolvaptan 120 mg per day or lanreotide 120 mg s.c. every 4 weeks above standard care.

Protein intake was calculated as 24-h urinary urea excretion * 0.18 + 15 according to the Maroni formula [17] and sodium intake as 24-h urinary sodium excretion. cAMP was measured in 24 h urine in a competitive protein-binding assay using a Radio Immuno Assay (Amersham plc, UK). GFR was estimated (eGFR) using the 2009 CKD-EPI (Chronic Kidney of Diet in Renal Disease) Study equation [18]. TKV was measured on T2-weighted coronal images using Analyze direct 9.0 (AnalyzeDirect, Inc., Overland Park, KS) with the manual tracing method and adjusted for height (htTKV) [19]. *PKD* mutation analysis was performed with DNA isolation using PUREGENE™ nucleic acid purification chemistry on the AUTOPURE LS 98 platform (Qiagen), followed by sequencing of amplified coding exons directly (exon 34–46), or on long-range PCR products (exon 1–33) [20].

### Somatostatin measurements

**Fig. 1:**
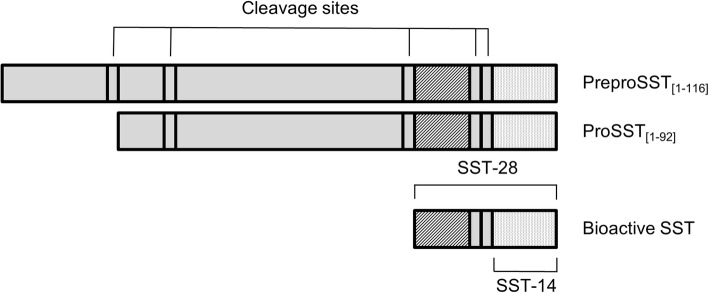
Somatostatin, its precursors and cleavage products (modified from chapter 9 of handbook of physiology by Patel et al. [26]). SST is synthesized as part of a large precursor protein, preproSST, located on the long arm of chromosome 3 and is rapidly cleaved into the prohormone form, proSST. ProSST is further enzymatically processed in two bioactive forms, SST-14 and SST-28 .These forms are both measured in the assay we used. Abbreviations are: SST, somatostatin

In this study we measured the concentration of bioactive SST (Fig. 1) in fasting blood samples. Therefore, blood was collected in EDTA tubes. All specimens were mixed by gentle hand inversion at least six times following collection. Samples were put on ice and separated immediately by centrifugation at 1580 g for 10 min at 4 degrees Celsius.

First, plasma samples were extracted on SEP-columns containing 200 mg of C18 (Strata, Phenomenex). The extracted samples were snap frozen by liquid nitrogen and thereafter stored at − 80 °C. Within 1 week after extraction, 3 mL of each sample was freeze dried in a Christ Epsilon 2–4 freeze dryer (Salm & Kipp, Breukelen, The Netherlands), at − 45 **°**C for 24 h at a pressure of 0.220 mbar and a condenser temperature of − 85 °C to remove water from the frozen samples. The dried extract was kept at − 80 **°**C until the ELISA took place. Within 1 week after freeze drying, the ELISA was performed by reconstitution of the dried extract, using 125 μL of assay buffer and 50 μL was aliquoted into designated assay wells using a commercially available ELISA kit (Phoenix pharmaceuticals Inc. Burlingame, California, USA). Concentrations were expressed as pg/mL. The intra- and inter-assay coefficient of variation (CV) of the SST ELISA were 2.4 and 7.6% respectively. The additional CV due to the extraction step with SEP-COLUMNS was 7.7%. Spiking with 50 and 100 pg SST-14 resulted in a median ratio of 1.14 (0.96–1.59) between measured and expected SST concentration. To minimize variability, multiple samples of an individual were assessed on one plate.

### Statistical analysis

Normally distributed data are expressed as means ± standard deviation, whereas non-normally distributed data are expressed as median with interquartile range (IQR). Differences between groups were tested using a 2-sample *t* test when normally distributed or a Mann-Whitney test when not normally distributed. Pearson Correlation was used to investigate the stability of SST measured twice over time.

Potential determinants of SST concentration were investigated using linear regression analysis. Associations with SST were tested univariably and multivariably in a stepwise backward analysis. Next, we investigated the association of SST concentration with urinary cAMP excretion, mGFR and htTKV cross-sectionally and with annual change in mGFR and htTKV longitudinally. We tested associations crude and after adjustment for covariates. Only patients with a follow-up time of ≥1 year were selected for the longitudinal analyses. Annual change in mGFR and htTKV were calculated as follow-up minus baseline value divided by follow-up time in years. SST was log transformed to fulfill the requirement of normal distribution of the residuals for the linear regression analyses. Outliers in SST were defined as values above or below two times the standard deviation of the mean of the log transformed SST values.

As sensitivity analysis we repeated the analysis with eGFR cross-sectionally and with annual change in eGFR calculated as slope longitudinally, with slope calculated by at least 3 eGFR measurements over > 1 year.

We compared SST levels in patients receiving standard care at baseline and standard care plus tolvaptan or lanreotide at follow-up using a Related-Samples Wilcoxon Signed Rank Test. A Mann-Whitney U test was performed to compare SST levels between the two groups.

Analyses were performed with SPSS version 23.0 (SPSS Inc., Chicago, IL). A two sided *p* < 0.05 was considered statistical significant.

## Results

### Patients and somatostatin concentration at baseline and over time

SST was measured in a total of 127 ADPKD patients for cross-sectional analyses. All patients had mGFR values and 121 patients had TKV values available. Baseline characteristics of patients are presented in Table 1.

**Table 1:** Baseline characteristics of ADPKD patients

	Standard care	Tolvaptan	Lanreotide
N	127	27	25
Age (yrs)	40.9 ± 11.0	46.3 ± 9.8	47.4 ± 9.7
Female, n (%)	56 (44.1)	12 (48.1)	11 (44.0)
BMI (kg/m^2^)	26.0 ± 4.6	25.7 ± 4.1	27.7 ± 5.4
BSA (m^2^)	2.03 ± 0.25	2.02 ± 0.28	2.04 ± 0.25
SBP (mm Hg)	128 ± 12.2	131 ± 10.7	140 ± 10.6
DBP (mm Hg)	78.7 ± 9.23	81.3 ± 7.6	88.1 ± 8.3
AHT n, (%)	99 (78.0)	24 (88.9)	22 (88.0)
Sodium intake (mmol/24 h)	170 ± 68.9	202 ± 92.7	168 ± 59.0
Protein intake (g/24 h)	85.7 ± 21.0	110 ± 38.5	67.8 ± 16.7
Coffee use (cups/day)	3 (1–5)	*NA*	*NA*
eGFR (ml/min/1.73m^2^)	72.5 ± 31.6	57.0 ± 33.1	51.0 ± 11.6
mGFR (ml/min/1.73m^2^)	75.2 ± 32.0	60.8 ± 34.7	*NA*
htTKV (mL/m)	826 (521–1297)	1242 (608–1505)	1150 (650–1833)
*PKD* mutation, n (%)			
*PKD1* truncating	48 (37.8)	3 (11.1)	9 (36.0)
*PKD1* non-truncating	30 (23.6)	5 (18.5)	8 (32.0)
*PKD2*	13 (10.2)	4 (14.8)	4 (16.0)
No mutation detected	2 (1.6)	0 (0.0)	0 (0.0)
Missing	34 (26.8)	15 (55.6)	4 (16.0)
Somatostatin (pg/mL)	48.5 (34.3–77.8)	38.2 (23.8–70.6)	42.5 (33.2–55.0)

Variables are presented as mean ± SD when normally distributed, when non-normally distributed as median (IQR)

*Abbreviations are*: *N* number, *BMI* body mass index, *BSA* body surface area, *SBP* systolic blood pressure, *DBP* diastolic blood pressure, *eGFR* estimated glomerular filtration rate, *mGFR* measured glomerular filtration rate, *htTKV* height adjusted total kidney volume, *PKD* polycystic kidney disease, *NA* not available

**Fig. 2:**
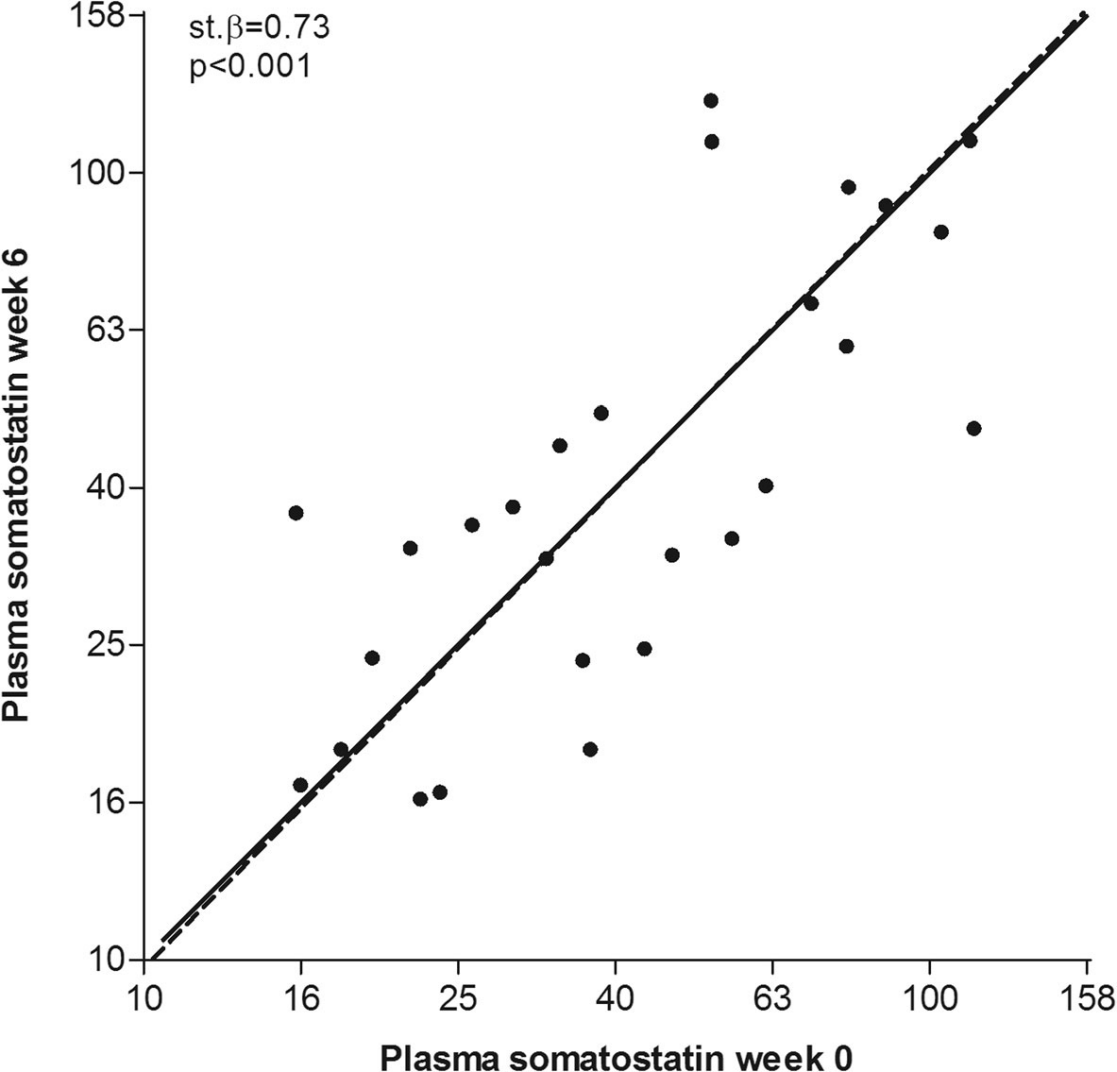
Correlation between plasma somatostatin at week 0 and week 6, with solid line representing the line of identity, and dotted line the actual regression line. The regression line is calculated by orthogonal linear regression analysis and standardized beta and *p*-value is calculated by Pearson Correlation

In a subset of 26 ADPKD patients, SST measurement was repeated during a follow-up of 42 ± 1 days. In this subset, SST levels were stable (40.8 (IQR 25.3–72.6; range 98.2) vs. 37.29 (IQR 24.67–72.12; range 107) pg/mL, *p* = 0.69), represented by Fig. 2 as the regression line follows the line of identity and showed a strong correlation (st. β = 0.73, *p* < 0.001).

### Possible determinants of somatostatin concentration at baseline

In ADPKD patients sex, age, protein intake and coffee use were univariably associated with SST concentration. When performing a multivariable stepwise backward analysis both age and protein intake remained associated with SST, indicating that these two variables are the most important determinants of SST concentration (Table 2).

**Table 2:** Potential determinants of SST concentration in ADPKD patients receiving standard care (*n* = 127)\

	Univariable		Multivariable Stepwise backward	
	St. β	*p*-value	St. β	*p*-value
Female sex	−0.20	0.02		
Age (yrs)	0.18	0.04	0.19	0.03
BMI (kg/m^2^)	−0.01	0.92		
AHT use	−0.06	0.48		
SBP (mmHg)	−0.04	0.70		
DBP (mmHg)	−0.05	0.60		
Sodium intake (mmol/24 h)	0.06	0.50		
Protein intake (g/24 h)	0.19	0.04	0.20	0.03
Coffee use (cups/day)	0.20	0.04		
*PKD2* (ref)^a^				
*PKD1* truncating	−0.10	0.53		
*PKD1* non-truncating	−0.09	0.59		

Standardized beta’s and *p*-values were calculated using linear regression analysis. Dependent variable is the log transformed SST concentration, independent variables are sex, age, BMI, AHT, SBP, DBP, sodium intake, protein intake and coffee use

*Abbreviations are: BMI* body mass index, *AHT* antihypertensive therapy, *SBP*, systolic blood pressure, *DBP* diastolic blood pressure, *PKD* polycystic kidney disease, *St. β* standardized beta

^a^*PKD* mutation was used as dummy variable with *PKD2* as reference group

### Somatostatin concentration and urinary cAMP at baseline

The mean urinary cAMP excretion was 3.89 ± 1.27 μmol/24 h. The level of cAMP excretion is, among others, dependent on the level of functioning kidney tissue (in our cohort, β = 7.18 and st. β = 0.34, *p* = 0.003 between cAMP excretion and mGFR). Therefore, we adjusted cAMP excretion for mGFR. Plasma SST was univariably associated with mGFR (adjusted urinary cAMP excretion) (β = 0.02 and st. β = 0.27, *p* = 0.02), however after adjustment for age and protein intake this association lost significance (β = 0.01 and st. β = 0.21, *p* = 0.09).

### Somatostatin concentration and disease severity at baseline

Table 3 shows the association of SST concentration with mGFR and htTKV. SST concentration was significantly associated with mGFR and htTKV. After adjustment for age and sex no association remained significant. Of note, also additional adjustment for renal function (in case of htTKV) or htTKV (in case of mGFR), and protein intake did not result in significant associations of SST with mGFR and htTKV. After excluding 4 outliers (subjects with a SST level of 1.05, 4.10, 9.50 and 343.67 pg/mL), SST remained associated with mGFR (β = − 29.0 and st. β = − 0.22, *p* = 0.01), but the association with htTKV lost significance (β = 0.09 and st. β = 0.08, *p* = 0.40). Of note, when excluding these 4 outliers, it was again noted that the association of SST with mGFR lost significance after adjustment for age and sex (β = − 16.3 and st. β = − 0.13, *p* = 0.09). Results with eGFR were similar compared to mGFR (Additional file 1). We found no differences in SST levels between CKD stages (Additional file 2).

**Table 3:** Association of plasma SST concentration with parameters of disease severity in patients receiving standard care

	Crude		Model 1		Model 2		Model 3		Model 4	
	St. β	p	St. β	p	St. β	p	St. β	p	St. β	p
Baseline mGFR (ml/min/1.73m^2^)										
Log SST	−0.25	0.004	−0.13	0.06	−0.10	0.14	−0.10	0.21	−0.12	0.14
Female sex			0.05	0.47	−0.02	0.75	−0.02	0.79	0.03	0.76
Age (yrs)			−0.59	< 0.001	−0.57	< 0.001	−0.63	< 0.001	−0.62	< 0.001
htTKV (ml/m)					−0.33	< 0.001	−0.34	< 0.001	−0.34	< 0.001
*PKD2* (ref)^a^										
*PKD1* truncating							−0.30	0.02	−0.29	0.02
*PKD1* non-truncating							−0.27	0.03	−0.26	0.03
Protein intake (g/24 h)									0.14	0.10
Baseline log htTKV										
Log SST	0.21	0.02	0.13	0.15	0.06	0.50	0.05	0.62	0.04	0.71
Female sex			−0.26	0.004	−0.23	0.004	−0.24	0.02	−0.21	< 0.05
Age (yrs)			0.17	0.05	−0.15	0.13	−0.16	0.22	−0.16	0.22
mGFR (ml/min/1.73m^2^)					−0.55	< 0.001	−0.56	< 0.001	−0.57	< 0.001
*PKD2* (ref)^a^										
*PKD1* truncating							−0.05	0.73	−0.05	0.74
*PKD1* non-truncating							−0.15	0.31	−0.15	0.31
Protein intake (g/24 h)									0.07	0.50

Standardized beta’s and *p*-values were calculated using linear regression analysis. Dependent variable is mGFR or log transformed htTKV, independent variable is the log transformed SST concentration

*Abbreviations* are: *SST* somatostatin, *mGFR* measured GFR, *htTKV* height adjusted total kidney volume, *PKD* polycystic kidney disease St. β, standardized beta, *p* p-value

Model 1: adjusted for age and sex

Model 2: adjusted for age, sex and htTKV or mGFR

Model 3: adjusted for age, sex and htTKV or mGFR and *PKD* mutation

Model 4: adjusted for age, sex and htTKV or mGFR, *PKD* mutation and protein intake

^a^*PKD* mutation was used as dummy variable with *PKD2* as reference group

### Somatostatin concentration and disease progression during follow-up

**Fig. 3:**
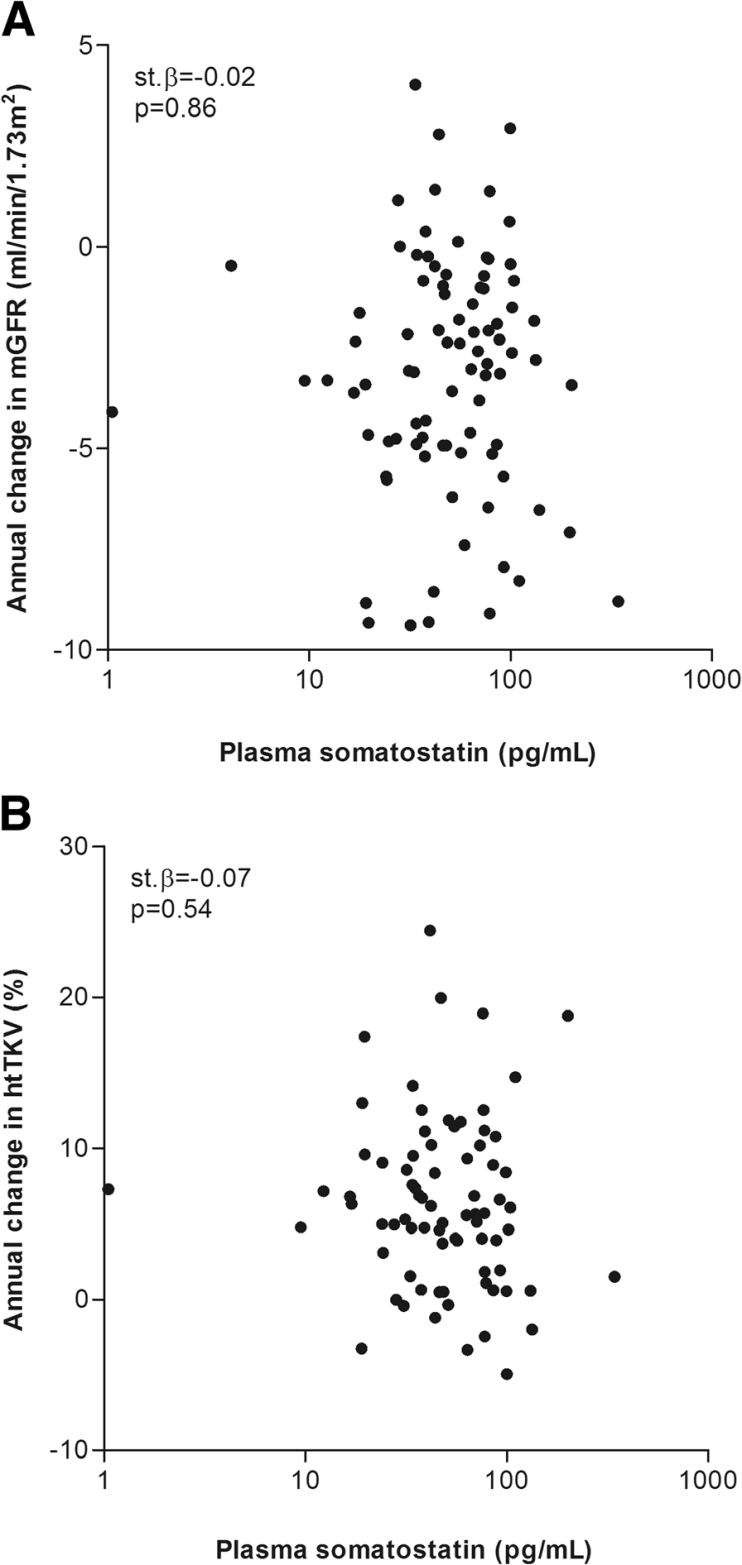
Association between baseline SST and annual change in mGFR (panel **a**) or annual change in htTKV (panel **b**) in patients with ADPKD receiving standard care. Standardized beta’s and *p*-values were calculated by linear regression analysis

Thirty patients were excluded for the longitudinal analyses because they were either lost to follow-up or had a follow up time < 1 year, leaving 97 patients. Seventy-eight patients had follow-up mGFR and 77 patients follow-up TKV data available. Mean follow-up time of these patients was 3.8 ± 1.3 years for mGFR and 3.8 ± 1.1 years for htTKV. The annual rate of mGFR decline was − 3.17 ± 2.99 ml/min/1.73m^2^ per year and the annual rate of htTKV growth was 6.37 ± 5.70% per year. Baseline SST concentration was not significantly associated with either annual change in mGFR or htTKV (β = − 0.17, st. β = − 0.02, *p* = 0.87 and β = − 1.18, st. β = − 0.07, *p* = 0.54 respectively) (Fig. 3). Of note, additional adjustment for age, sex, baseline htTKV (in case of annual change in mGFR), baseline mGFR (in case of annual change in htTKV), *PKD* mutation and protein intake did not materially change these associations (β = 0.66, st. β = 0.07, *p* = 0.51 and β = − 2.08, st. β = − 0.12, *p* = 0.32, respectively). Excluding 4 outliers (subjects with a SST level of 1.05, 4.10, 9.50 and 343.67 pg/mL) did not change the results. Similar results were found when annual change in eGFR calculated as slope instead of annual change in mGFR was studied (Additonal file 1).

### Somatostatin concentration during tolvaptan or lanreotide therapy

**Fig. 4:**
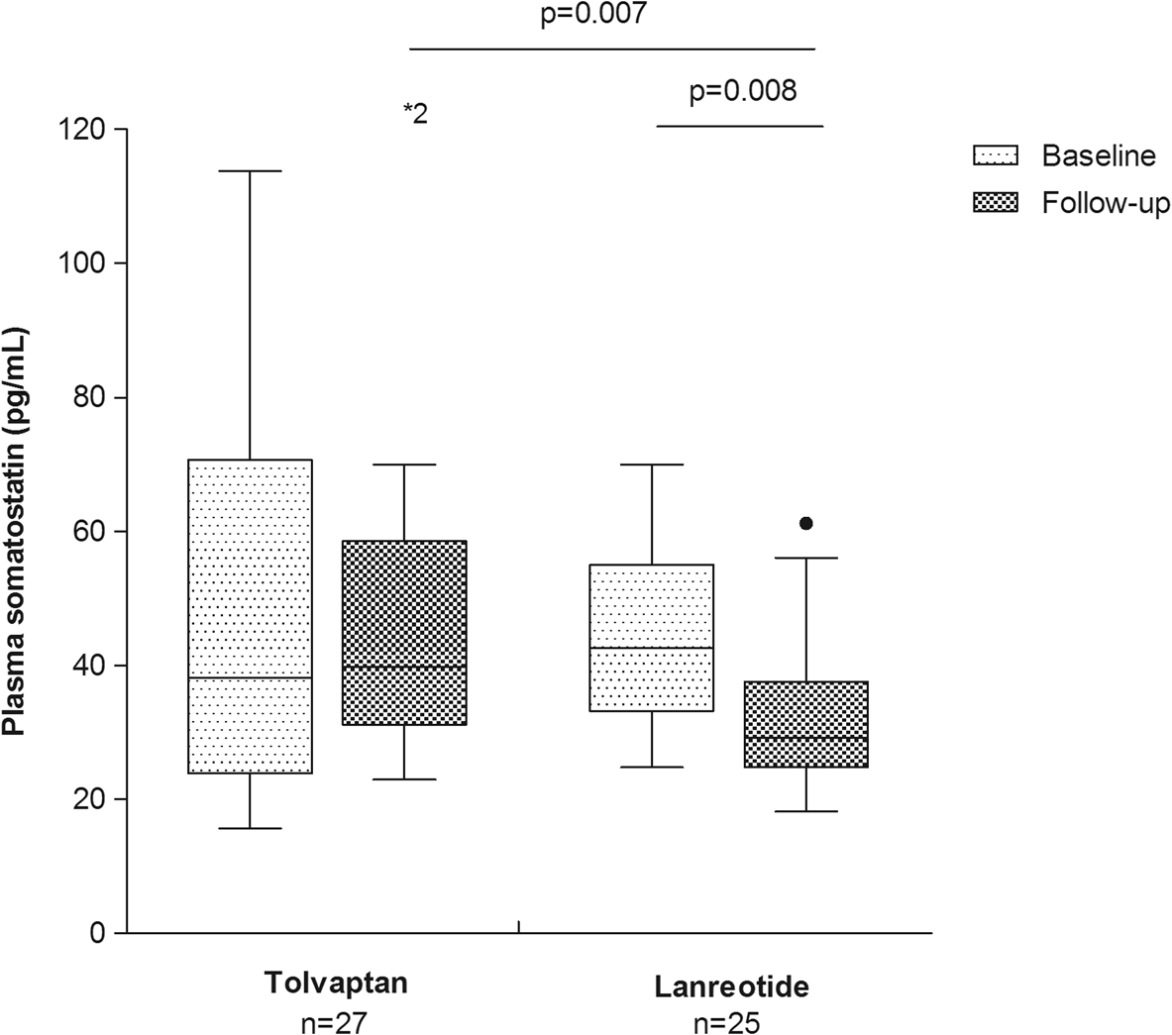
Plasma somatostatin concentration of patients receiving standard care at baseline and either tolvaptan or lanreotide above standard care at follow-up. Data are expressed as Tukey boxplots with median, IQR, and minimum and maximum within 1.5 IQR and outliers. * Indicate outliers outside the graph. *P*-values were calculated with a Related-Samples Wilcoxon Signed Rank Test in case of dependent measurements, and a Mann-Whitney U test in case of independent measurements

We compared SST levels of ADPKD patients receiving standard care at baseline and either tolvaptan (*n* = 27) or lanreotide (*n* = 25) above standard care at follow-up. These two groups consisted of patients with a similar age (46.3 ± 9.8 vs. 47.4 ± 9.7 years, *p* = 0.69), sex distribution (48% vs. 44% female, *p* = 0.79), eGFR (57.0 ± 33.1 vs. 50.9 ± 11.5 ml/min/1.73m^2^, *p* = 0.38) and htTKV (1242 (608–1504) vs. 1150 (650–1833) ml/m, *p* = 0.75) (Table 1). In patients receiving tolvaptan, SST levels remained stable (38.2 (IQR 23.8–70.7; range 98.2) pg/mL vs. 39.8 (IQR 31.2–58.5; range 295) pg/mL, *p* = 0.85), whereas in patients receiving lanreotide, SST levels decreased (42.5 (IQR 33.2–55.0; range 45.3) pg/mL vs. 29.3 (IQR 24.8–37.6; range 43.0) pg/mL, *p* = 0.008) (Fig. 4). SST levels of both groups were similar at baseline (*p* = 0.87) but lower in patients receiving lanreotide at follow-up (*p* = 0.007).

## Discussion

In the present study, we did not find an association between SST and urinary cAMP excretion, disease severity or disease progression. This suggests that plasma SST is not involved in the pathophysiology of ADPKD. However, we did find lower levels of plasma SST during treatment with lanreotide, whereas plasma SST levels remained stable during treatment with tolvaptan.

An important determinant of plasma SST is nutrition [21]. To ensure optimal standardization, we drew fasting blood samples for the measurement of SST. We subsequently investigated if there were determinants of plasma SST in our study population and found that univariably sex, age, protein intake and coffee use were significantly associated with SST. Both age and protein intake remained associated with SST in the multivariable stepwise backward analysis, indicating that these variables may be determinants of plasma SST concentration. That age is associated with plasma SST concentration, with higher SST levels with increasing age, is confirmed by other studies that investigated SST in relation to growth hormone concentration [22–25]. The exact mechanism why SST increases with age is not completely understood, but there is evidence that this is related to changes in sex- and growth hormone levels [23]. The association of protein intake with plasma SST concentration can be explained since ingestion of proteins gives rise to gastrin release, that in turn stimulates SST secretion [21]. Accordingly, we adjusted all our further analyses for age and protein intake.

Only limited data is available on plasma concentration of endogenous SST and its physiological effects on the kidney. To our knowledge this is the first study investigating associations of systemic plasma concentration of SST in relation to a kidney disease, specifically ADPKD. The results lead us to reject our hypothesis that plasma SST is associated with urinary cAMP excretion, disease severity and disease progression in ADPKD. An explanation might be the balance between plasma SST concentration and SST receptor affinity. There are five SST receptor subtypes (SST receptor 1–5) and SST-14 and SST-28 have for instance an affinity for the SST receptor 1 between 0.1 and 2.26 nM and for the SST receptor 2 between 0.2 and 4.1 nM, with affinity expressed as the concentration required for obtaining 50% of the maximum effect mediated by the receptor (EC50) [21]. Importantly, the median fasting SST concentration in our ADPKD cohort was 48.5 (34.3–77.8) pg/mL, which is equivalent to approximately 0.03 nM, much lower than these EC50’s. Of note, these concentrations are comparable to that found in healthy subjects in other studies (14–32.5 pg/ml) [26]. Our findings suggest that systemic plasma SST concentration is not involved in the pathophysiology of ADPKD. However, this does not necessarily deny a role for SST in the pathophysiology of ADPKD. SST is mainly produced at local sites of action, thereby eliciting a paracrine/autocrine action. For example for the stimulation of growth hormone, SST is released from cells in the hypothalamus adjacent to the cells that secrete growth hormone, and for the inhibition of pancreatic insulin secretion, the δ cell in the pancreas releases SST. A similar mechanism has been suggested for the kidney; in-vitro studies showed that mesangial cells and proximal tubular cells itself produce SST [6, 27]. This suggests that circulating concentrations of SST, that probably consist mainly of SST secreted from the gastro-intestinal tract [28–31], do not reflect the SST concentrations locally at tissue level for instance in the kidney.

By its paracrine/autocrine property, SST can elicit an organ specific action, despite its broad systemic effects on various SST receptors. To allow such organ specific actions, SST is rapidly inactivated following local release by peptidases in blood, but also by peptidases at tissue level, thereby minimizing unwanted systemic effects. Exogenous SST analogues, that are used as therapeutic agents, elicit their specific effects by having more affinity for one receptor than for the other. Furthermore, these SST analogues have a considerably longer half-life than endogenous SST, which makes these SST analogues clinically applicable as a drug, in contrast to endogenous SST.

Lanreotide, which is a SST analogue, has nanomolar affinity for only SST receptor 2 (EC50: 0.5–1.8 nM) and SST receptor 5 (EC50: 0.6–14 nM). These receptors are predominantly expressed in the brain, gastro-intestinal tract and kidneys, while endogenous SST-14 and SST-28 have nanomolar affinity for all of the five receptor subtypes. Furthermore, lanreotide has a plasma half-life of 25.5 days which is much longer than that of endogenous SST [21, 32]. Therapy with lanreotide 90 mg reaches mean plasma concentrations of 4455 pg/mL [21, 32], which is equivalent to approximately 2.7 nM. The renal SST receptors can easily be triggered by these concentrations of lanreotide and elicit effects involved in inhibiting disease progression in ADPKD. This is currently being investigated in several randomized clinical trials [14]. In the present study we showed in a small subset of included ADPKD patients, that plasma levels of endogenous SST decrease during treatment with lanreotide. It may be that this decline in plasma SST levels during administration of lanreotide reflects the extent to which SST receptors are triggered, and thus indirectly reflects efficacy of lanreotide treatment in ADPKD patients. This exciting option has to be investigated in prospective studies. We hypothesized that during treatment with the vasopressin V2 receptor antagonist tolvaptan, SST levels may also change. It is known that SST can inhibit cAMP production but cAMP in turn can stimulate SST secretion [21]. Furthermore, it is suggested that SST has a modulating effect on diuresis [33–36]. Since tolvaptan can effectively slow the rate of disease progression in ADPKD by inhibiting renal cAMP production [10, 11] and stimulate diuresis by blocking vasopressin at the collecting duct, SST levels could theoretically be influenced during treatment with tolvaptan. However, we found no differences in systemic SST levels during treatment with tolvaptan. Since tolvaptan acts locally in the collecting duct of the kidney it could again be that only locally produced SST levels change during treatment with tolvaptan.

It should be noted that the present study has a number of limitations. First, we used biobanked blood samples that were collected without exogenous protease inhibitors, whereas some have suggested that the use of such inhibitors leads to more reliable assessment of SST levels. Moreover, we found relatively high inter-assay CV’s of the SST measurement. However, we found an excellent yield of measured and expected SST concentration in plasma samples spiked with exogenous SST (ratio 1.14 (0.96–1.59)). In addition, we found a strong correlation between SST levels measured twice in a subset of ADPKD patients (Fig. 3). We conclude therefore that the samples and assay are sufficiently reliable to detect associations and changes in SST within a patient. Strengths of our study are that this is the first study investigating plasma SST in relation to a renal disease. Second, we had detailed follow-up data available for our patients, with gold standard measurements of disease progression (change in mGFR and TKV) enabling us to investigate longitudinal associations of baseline plasma SST with the rate of disease progression. We also investigated change in plasma SST levels during treatment with the vasopressin V2 receptor antagonist tolvaptan and the SST analogue lanreotide.

## Conclusions

This study demonstrates that fasting plasma concentration of SST was not associated with urinary cAMP excretion, disease severity or disease progression in patients with ADPKD.

## Additional files

Additional file 1: Multivariable linear regression analysis of the association of SST concentration with eGFR at baseline (*n* = 127) (upper panel) or annual change in eGFR calculated as slope through multiple (≥3) eGFR values at follow-up (*n* = 97) (lower panel). (DOCX 24 kb)

Additional file 2: Somatostatin levels according to CKD stage in patients receiving standard care. Data are expressed as Tukey boxplots with median, IQR, and minimum and maximum within 1.5 IQR and outliers (TIF 381 kb)

## Abbreviations

ADPKD: Autosomal dominant polycystic kidney disease
cAMP: Cyclic adenosine monophosphate
CV: Coefficient of variation
eGFR: Estimated glomerular filtration rate
htTKV: Height adjusted total kidney volume
IQR: Interquartile range
mGFR: Measured glomerular filtration rate
SST: Somatostatin
