# Supplementary material for: Association of plasma somatostatin with disease severity and progression in patients with autosomal dominant polycystic kidney disease
Source: BMC Nephrol. 2018 Dec 19;19:368. doi: 10.1186/s12882-018-1176-y (PMC6299932; doi:10.1186/s12882-018-1176-y)
Supplement: Supplementary file 1 — Multivariable linear regression analysis of the association of SST concentration with eGFR at baseline (n = 127) (upper panel) or annual change in eGFR calculated as slope through multiple (≥3) eGFR values at follow-up (n = 97) (lower panel). (DOCX 24 kb) [file 12882_2018_1176_MOESM1_ESM.docx]

**Additional file 1:** Multivariable linear regression analysis of the association of SST concentration with eGFR at baseline (n=127) (upper panel) or annual change in eGFR calculated as slope through multiple (≥3) eGFR values at follow-up (n=97) (lower panel).

|  |  | **Crude** | |  | **Model 1** | |  | **Model 2** | |  | **Model 3** | |  | **Model 4** | |
| --- | --- | --- | --- | --- | --- | --- | --- | --- | --- | --- | --- | --- | --- | --- | --- |
|  |  | **R** | **p** |  | **St. β** | **p** |  | **St. β** | **p** |  | **St. β** | **p** |  | **St. β** | **p** |
| **Baseline eGFR (ml/min/1.73m^2^)** |  |  |  |  |  |  |  |  |  |  |  |  |  |  |  |
| Log SST |  | -0.18 | <0.05 |  | -0.08 | 0.28 |  | -0.05 | 0.49 |  | -0.05 | 0.54 |  | -0.07 | 0.41 |
| Female sex |  |  |  |  | -0.05 | 0.49 |  | -0.12 | 0.09 |  | -0.12 | 0.14 |  | -0.08 | 0.39 |
| Age (yrs) |  |  |  |  | -0.60 | <0.001 |  | -0.57 | <0.001 |  | -0.64 | <0.001 |  | -0.62 | <0.001 |
| htTKV (ml/m) |  |  |  |  |  |  |  | -0.31 | <0.001 |  | -0.33 | <0.001 |  | -0.33 | <0.001 |
| *PKD2* (ref)^$^ |  |  |  |  |  |  |  |  |  |  |  |  |  |  |  |
| *- PKD1* truncating |  |  |  |  |  |  |  |  |  |  | -0.33 | 0.01 |  | -0.32 | 0.01 |
| *- PKD1* non-truncating |  |  |  |  |  |  |  |  |  |  | -0.30 | 0.01 |  | -0.30 | 0.01 |
| Protein intake (g/24hr) |  |  |  |  |  |  |  |  |  |  |  |  |  | 0.13 | 0.14 |
| **Annual change in eGFR (ml/min/1.73m^2^)** |  |  |  |  |  |  |  |  |  |  |  |  |  |  |  |
| Log SST |  | -0.05 | 0.64 |  | -0.07 | 0.47 |  | -0.02 | 0.89 |  | -0.00 | 0.98 |  | 0.03 | 0.80 |
| Female sex |  |  |  |  | 0.01 | 0.95 |  | 0.06 | 0.59 |  | 0.05 | 0.64 |  | -0.11 | 0.32 |
| Age (yrs) |  |  |  |  | 0.15 | 0.15 |  | 0.30 | 0.02 |  | 0.09 | 0.54 |  | 0.08 | 0.61 |
| eGFR (ml/min/1.73m^2^) |  |  |  |  |  |  |  | 0.16 | 0.22 |  | 0.05 | 0.73 |  | 0.08 | 0.56 |
| htTKV (ml/min) |  |  |  |  |  |  |  | -0.35 | 0.002 |  | -0.40 | <0.001 |  | -0.38 | 0.001 |
| *PKD2* (ref)^$^ |  |  |  |  |  |  |  |  |  |  |  |  |  |  |  |
| *- PKD1* truncating |  |  |  |  |  |  |  |  |  |  | -0.51 | 0.003 |  | -0.52 | 0.002 |
| *- PKD1* non-truncating |  |  |  |  |  |  |  |  |  |  | -0.34 | 0.04 |  | -0.34 | 0.04 |
| Protein intake (g/24hr) |  |  |  |  |  |  |  |  |  |  |  |  |  | -0.19 | 0.09 |
| Standardized beta’s and p-values were calculated using linear regression analysis. Dependent variable is either eGFR or annual change in eGFR and the independent variable is the log transformed SST concentration. Model 1: adjusted for age and sex Model 2: adjusted for age, sex and baseline htTKV (and eGFR)  Model 3: adjusted for age, sex, baseline htTKV, (baseline eGFR) and *PKD* mutation Model 4: adjusted for age, sex, baseline htTKV, (baseline eGFR), *PKD* mutation and protein intake ^$^ *PKD* mutation was used as dummy variable with *PKD2* as reference group;  *Abbreviations* are: eGFR, estimated GFR; St. β, standardized beta; p, p-value; htTKV, height adjusted TKV; *PKD*, polycystic kidney disease; SST, somatostatin. | | | | | | | | | | | | | | | |
