# Supplementary figures and images for: Association of plasma somatostatin with disease severity and progression in patients with autosomal dominant polycystic kidney disease
Source: BMC Nephrol. 2018 Dec 19;19:368. doi: 10.1186/s12882-018-1176-y (PMC6299932; doi:10.1186/s12882-018-1176-y)

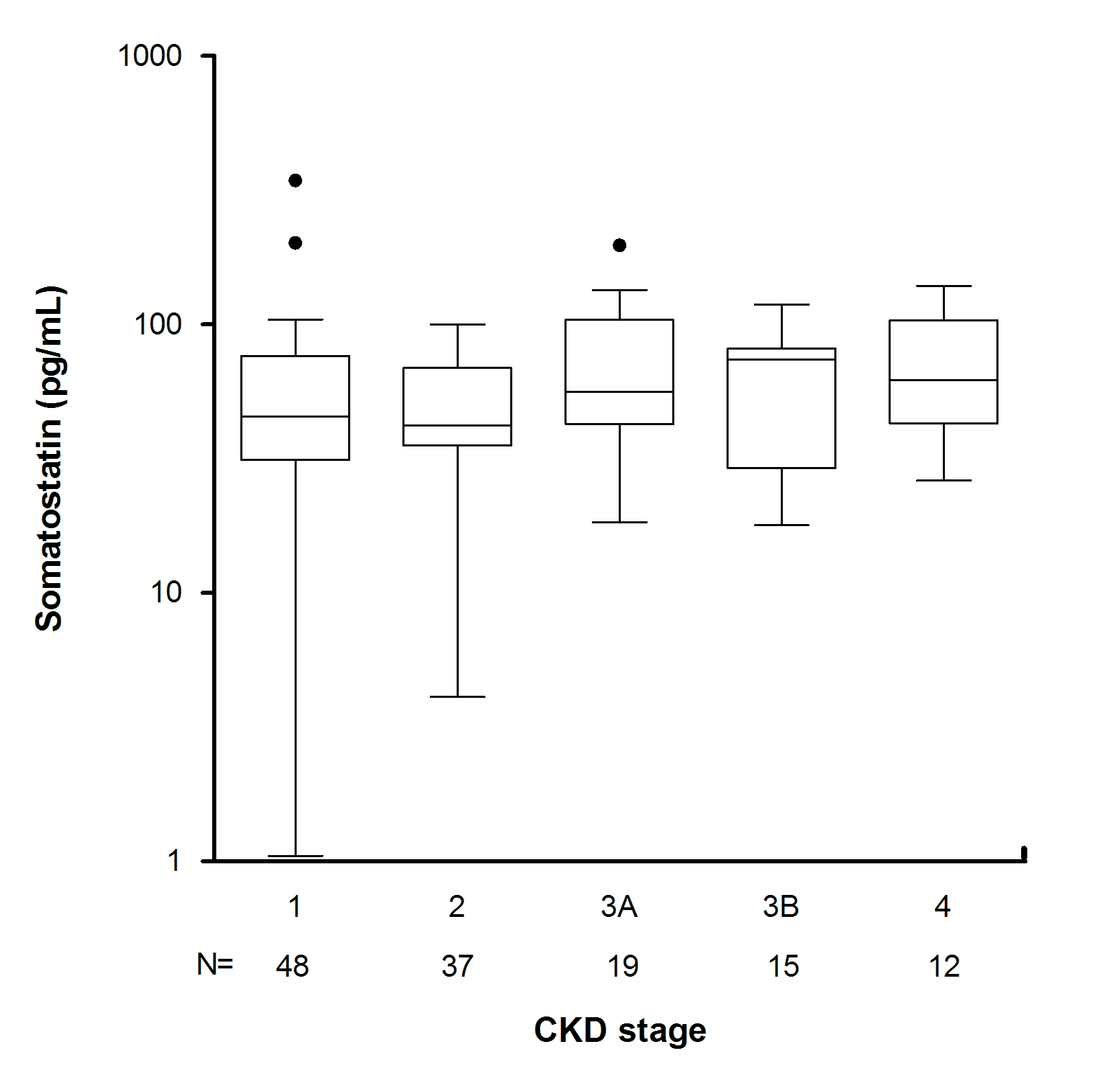

Supplement: Supplementary file 2 — Somatostatin levels according to CKD stage in patients receiving standard care. Data are expressed as Tukey boxplots with median, IQR, and minimum and maximum within 1.5 IQR and outliers (TIF 381 kb) [file 12882_2018_1176_MOESM2_ESM.tif]
